# Supplementary material for: The role of sex education in withdrawal use: Prevalence and correlates among a nationally representative sample of adolescents and young adults
Source: PLoS One. 2026 Jan 16;21(1):e0340279. doi: 10.1371/journal.pone.0340279 (PMC12810779; doi:10.1371/journal.pone.0340279)
Supplement: Attachment — Submitted filename: Responses to Editor and Reviewers Comments.docx [file pone.0340279.s003.docx]

| Editor comments |  |
| --- | --- |
| Please define the age group of youth as there are few differences in definitions globally. | We are delighted for the opportunity to revise and resubmit our manuscript to *PLOS ONE*. We have addressed each of the Reviewers’ comments by either making a change/edit in the revised manuscript or providing further rationale for our original decision (outlined in detail below). As a result of the Reviewer comments, we believe the revised manuscript has been greatly strengthened and we hope that you agree. Please do not hesitate to let us know if you have any questions or if you require additional changes/edits. Thank you in advance. We look forward to hearing back from you!  Regarding age definitions of youth: Thank you for pointing this out! This comment has been addressed, information has been added to the introduction and methods section. |
| Does the national survey capture the socioeconomic status in the national survey? This could be a valuable covariate! | This is an excellent question. While this may be a valuable covariate, this was not the focus of our present study. Based on a different reviewer comment, however, additional information was added to the limitations section regarding what covariates were included or excluded. |
| Lines 40-42-Abstract; Conclusion- Please reword | This comment has been addressed. |
| Line-67- reference at completion of the sentence! | This comment has been addressed. |
| Lines 77-81- Please reword the sentence! | This comment has been addressed. |
| Lines 87-89- Purpose does not match with the title and the objective given in the abstract! Please consider using “youth”. | This comment has been addressed. |
| Line 102- Please explain the reason for exclusion of pregnant youth. Specially with questions like “define withdrawal use: 1) ever use, 2) at first sex, 3) past 12 months, and 4) 109 past 3 months.[24]” | Thank you for this comment. We decided to exclude pregnant youth to get an unbiased sample of those using a form of contraception regularly and without having been pregnant before. |
| Table 1 one- Will it possible to compare the demographic with national US data? | The NSFG collects data using computer-assisted personal interviews of men and women aged 15-49 years in the United States. The NSFG utilizes a stratified, multistage, clustered, probability sampling design to report nationally representative estimates, meaning that the survey is designed to be nationally representative of men and women aged 15-49 living in households in the United States. As these data are already nationally representative, we feel there is no added value to report duplicate demographic characteristics in the table. |
| Table 1- Education level could be better presented with breakdown of the youth group say 15-19 and above 19 which could reduce the misinterpretation of higher representation of 9th grade or less/ high school in study cohort. | Thank you for this suggestion. We feel that would misrepresent the data since the item from the NSFG asks about the *highest* education level respondents reported receiving in their life (not what level of education they are currently receiving or are currently in). |
| Table 2 & 4- It is better if you could present the survey wave results (overall) for the four variables in graph format for better presentation! The 1st 4 rows of the table 2. | Thank you! We have removed the wave prevalence rows in tables 2 and 4 and have added two graphs depicting these prevalences over time for each outcome at each timepoint. Please see the revised Figures 1 and 2. |
| Lines 190-192- Discussion- It is important that you explore the reasons for comparatively higher prevalence in 3rd wave and subsequent drop in 4th wave. This is an important discussion point! | After further review, we agree. We have added additional information to the discussion section to address this comment (please see lines 222-225). |
| Lines199-205- What is the influence of social media/ online content in determining the contraceptive practice! | Additional information has been added throughout the manuscript. |
| Reviewer 1 |  |
| Review of manuscript PONE-D-24-14716 entitled "Prevalence and correlates of withdrawal as contraceptive method in a nationally representative sample of youth: A cross-sectional study". The manuscript examines the trend of withdrawal use (exclusively or in combination with other methods) and its relation with the receipt of sexual education. The results contribute to the literature. The following issues need to be addressed before it can be publishable. | Thank you for taking time to review our manuscript. Your feedback has strengthened our manuscript, and we are eager for the follow-up review. |
| - P 4, LL 66-67; 73-74: The authors discuss that withdrawal is less effective method, while the failure rate of "correct" and "consistent" use of withdrawal is 4%. The higher failure rate of withdrawal is referred to its "typical use" that is estimated to be 20% in the USA and more or less in other parts of world (suggest reading this paper https://doi.org/10.1038%2Fs41598-023-37398-1). The authors need to specify that they are talking about the failure rate of the typical use of withdrawal. Studies on withdrawal usually tend to undermine the importance of this method for women's health (no side effects) and its wide availability, and its role in fertility transition. | This comment had been addressed and additional information has been added. |
| - P. 5, LL83-84: The literature shows that the internet is the main source of sexuality education rather parents and siblings. | This comment has been addressed. |
| - Introduction: the importance of studying the relationship between sexuality education and withdrawal use has not been well stated. As mentioned, withdrawal is not part of family planning and sexual formal education of health systems. So, studying its relationship with withdrawal use does not sound clear to me here. The review of literature on this topic is very limited and short in this manuscript. | This comment has been addressed. |
| - L. 105: "Primary Measures" is an unusual term used for "dependent variables). Please consider revising it to "outcome" or "dependent" variables. | This comment has been addressed. |
| - There is a lack of theoretical logic for selecting covariates. Except the measures of sexuality education, al demographic variables have been studied by past studies. They are not the determinants of withdrawal use. I would suggest reviewing a tick literature on the relationship between sexuality education, as the source of contraceptive knowledge, and contraceptive use, which makes a good background for studying this relationship. | This comment has been addressed. |
| - L. 135: Please specify the type of logistic regression used and the justification for its use. | Thank you for the request for clarification! We have added more detail in lines 165-168 of the revised manuscript to address this comment. |
| - The findings in LL. 171-174 is one of the key results of this study that needs an explanation in Discussion section. Please make sure to expand the discussion of why the receipt of informal sexuality education related to other methods of birth control was associated with the greater risk of using withdrawal in LL  198-202. Can this be due to the fact that those receiving education on the side effects of modern birth control (especially pills) are more likely to use withdrawal with combination of condoms in order to avoid the side effects of Pills and other hormonal methods? | This comment has been addressed. |
| - L 194: Revise " One potential reason for reason for". | This comment has been addressed. |
| Reviewer #2 |  |
| Thank you for the opportunity to review this manuscript. I appreciate the authors' efforts in conducting this study which explores an important area. The manuscript shows promise but requires further development. The research question is interesting, but the discussion could be strengthened. Minor revisions are recommended. Please see specific comments below. | Thank you for taking the time to review the manuscript. We appreciate your thorough review and believe the addressed edits have strengthened the manuscript. |
| Abstract  Results:  •The author mentions the prevalence of withdrawal is higher in combination than alone (lines 34-35). I would suggest adding “across all waves and reference period” and accompanying it with values to improve clarity.  •Suggest to report values for outputs from the regression model; AOR with CI  Conclusion:  broad and ambiguous. It could be precise and specific. | We thank the reviewer for these comments. We have added the suggested blurb as well as the statistical results into the revised abstract. Please see the revised manuscript with track changes (lines 34-38). |
| Main Manuscript  Methods:  Line 127- formal sexual education: it would be useful to mention the various sources similar to the types of informal sources mentioned. | This comment has been addressed throughout the manuscript. |
| Results:  •Line 166 refers to Table 3, it should be Table 4 | This comment has been addressed. |
| •Table 4: Prevalence of non-Hispanic other population under ever use is reported incorrectly | Thank you for catching this error. We have corrected it in the table. |
| Discussion:  •The findings of this study are based on contraceptive use that dates back to 5 years ago. The pattern of contraceptive use likely changed since the data collection for this study. If the finding were to be relevant even today to draw meaningful conclusions based on which policy decisions are made, it would be useful to compare patterns of contraceptive use from recent literature in the absence of updated prevalence rates of withdrawal in particular. This could strengthen the findings of the current study although not updated. | The most recent NSFG data is the 2017-2019 cohort. However, recent literature has been added throughout the manuscript to strengthen our findings. |
| •A great many important covariates that have a direct influence on contraceptive use have not been explored in the study which is a major limitation and requires mentioning. Ex: previous experience, marital status, women's concerns on side effects preventing them from accessing other birth control methods, male partners' influence on deciding, power dynamics within relationships, educational level of the partner, cultural norms, economic status and cost of health insurance cover, sexual behaviour etc. However, given that the study was based on secondary data which would have failed to capture crucial factors, it would be useful to discuss the potential influence of these factors on the findings. | Additional information has been added to our limitations section. |
| •Prevalence alone or in combination with other methods increased from 2011 to 2017 and then declined across all reference periods except for the 2013-2015 wave at first sex (Table 2). This trend observed needs an explanation. | We have added more information to try to explain this trend. |
| •Line 199-202: The authors' explanation for higher odds of withdrawal used in combination with other methods is ambiguous. A possible explanation would be a sense of safety perceived when combined with other more effective methods. It still fails to explain the continuation of withdrawal despite being better informed. | This comment has been addressed, and additional information and insights have been added throughout the discussion section. |
| •Line 211 needs a reference for this claim. | This comment has been addressed. |
