# Supplementary material for: The role of sex education in withdrawal use: Prevalence and correlates among a nationally representative sample of adolescents and young adults
Source: PLoS One. 2026 Jan 16;21(1):e0340279. doi: 10.1371/journal.pone.0340279 (PMC12810779; doi:10.1371/journal.pone.0340279)
Supplement: Attachment — Submitted filename: Reviewer commnets for the revised manuscript.docx [file pone.0340279.s002.docx]

The authors have addressed most of the comments adequately. However, the following still needs to be addressed

1. Abstract- results- although prevalence has been reported, it is best reported accompanied with Confidence intervals. (Interval estimates).
2. The original comment “Methods**: Line 127- formal sexual education: it would be useful to mention the various sources similar to the types of informal sources mentioned”**. - not addressed, although the authors say they have! Line 157-159 in the revised manuscript.
