# Supplementary material for: The role of sex education in withdrawal use: Prevalence and correlates among a nationally representative sample of adolescents and young adults
Source: PLoS One. 2026 Jan 16;21(1):e0340279. doi: 10.1371/journal.pone.0340279 (PMC12810779; doi:10.1371/journal.pone.0340279)
Supplement: Attachment — Submitted filename: Response to Reviewer Comments.docx [file pone.0340279.s004.docx]

| Reviewer 2 | Author Response |
| --- | --- |
| The authors have addressed most of the comments adequately. However, the following still needs to be addressed: | Thank you! |
| Abstract- results- although prevalence has been reported, it is best reported accompanied with Confidence intervals. (Interval estimates). | As this was not a statistical comparison, we have added the individual standard errors to accompany the prevalences. This comment has been addressed. |
| The original comment “Methods**: Line 127- formal sexual education: it would be useful to mention the various sources similar to the types of informal sources mentioned”**. - not addressed, although the authors say they have! Line 157-159 in the revised manuscript. | To fully respond to the reviewer comment, additional information has been added to the introduction (lines 91-94) and methods section (line 146-147). |
| Reviewer 3 | Author Response |
| Thank you for the opportunity to review this revised manuscript – PONE-D-24-14716_R1. Most of the editor comments and two reviewers’ comments were addressed. Recommend to publish with the following minor revisions. | Thank you for taking the time to review the manuscript. We appreciate your thorough review and believe the addressed edits have strengthened the manuscript. |
| Defining the age group of youth should be clearer and should be unique throughout the manuscript. | In response to the reviewer comment, we have deleted our use of the term “Youth”, as definitions vary across the globe, and replaced it with a more current and accurate reference to young people this age, “Adolescents and young adults” or AYAs. This change has been made throughout the manuscript, including in the title, abstract, and table headers. |
| In the line 66 – put the reference at the completion of the sentence. | This comment has been addressed. We have also “cleaned up” the citations throughout the revised manuscript, ensuring consistency and adherence to journal guidelines. |
| Lines 88 – 90 consider using “youth” still not addressed properly. | Thank you for drawing our attention to this oversight. As noted above, we have deleted our use of the term “Youth” and replaced it with a more current and accurate reference to young people this age, “Adolescents and young adults” or AYAs. This change has been made throughout the manuscript, including in the title, abstract, and table headers. |
| Reviewer 4 | Author Response |
| **Reviewer #4**: This was an interesting article researching a currently relevant topic. Congratulations on a job well done! I have three concerns, as all others have been addressed by previous reviewers. | Thank you! |
| My first concern is in the discussion section line 213-216, where you describe "the increase of withdrawal use during the 2015-2017 wave may be partially explained by the beginning of the Donald Trump administration which heavily defunded comprehensive sexual health programs between 2016 and 2020". - the timing for the explanation does not line up as the change happens before the administration comes into power. Therefore, your conclusion loses it's validity. | Thank you for drawing our attention to this! This comment has been addressed (line 214-221). |
| My second cencern is the clain that this is an "update". line 195-197. "The goal of the current study was to provide an update of the prevalence of withdrawal as a contraceptive method in a nationally representative sample of American youth as well as to examine the relationship between informal sexuality education and withdrawal". - as the most recent data is from 2019, you may need to clarify regarding the availability of the data. That is, the most recent available NSFG data is from ........ | The comment has been addressed. |
| My third concern is that the persons delivering formal sexual education have not been specified or identified. That is, the receipt/ acceptance of sexual education may differ on the expertise/ perceived reliability of the individual delivering it. Thus it would be great to have it addressed in the writing.). | This comment has been addressed. Based on a previous reviewer comment, additional information has been added to the introduction and methods section to give a clear definition of where formal sexual education may have been received (see lines 91-94, as an example. |
